# Reactive Oxygen Species in the Tumor Microenvironment: An Overview

**DOI:** 10.3390/cancers11081191

**Published:** 2019-08-16

**Authors:** Frank Weinberg, Nithya Ramnath, Deepak Nagrath

**Affiliations:** 1 Division of Hematology and Oncology, Department of Medicine, University of Michigan, Ann Arbor, MI 48109, USA; 2 VA Ann Arbor Health Care System, Ann Arbor, MI 48109, USA; 3 Department of Biomedical Engineering, University of Michigan, Ann Arbor, MI 48109, USA; 4 Biointerfaces Institute, University of Michigan, Ann Arbor, MI 48109, USA; 5 Department of Chemical Engineering, University of Michigan, Ann Arbor, MI 48109, USA

**Keywords:** ROS, mitochondria, tumorigenesis, tumor microenvironment, stroma, tissue infiltrating lymphocytes, metabolism

## Abstract

Reactive oxygen species (ROS) are important signaling molecules in cancer. The level of ROS will determine physiological effects. While high levels of ROS can cause damage to tissues and cell death, low levels of ROS can have a proliferative effect. ROS are produced by tumor cells but also cellular components that make up the tumor microenvironment (TME). In this review, we discuss the mechanisms by which ROS can affect the TME with particular emphasis on tumor-infiltrating leukocytes. Greater insight into ROS biology in this setting may allow for therapeutic manipulation of ROS levels in order to remodel the tumor microenvironment and increase anti-tumor activity.

## 1. Introduction

Reactive oxygen species (ROS) include superoxide, hydrogen peroxide and hydroxyl radicals. While ROS can be damaging to lipids, proteins and DNA, in recent years their role as important intracellular and extracellular signaling molecules has become evident [1]. The mitochondria are the major source of ROS within a cell and play an essential role in regulation of proliferative, apoptotic and metabolic pathways [2,3,4]. It is established that the hallmarks of cancer include metabolic reprogramming as well as a tumor promoting microenvironment [5]. At the interface of both of these important biological events are ROS which are produced by cancer cells as well as cellular components in the microenvironment [6,7,8,9]. Understanding how the crosstalk between both extracellular and intracellular ROS not only within the tumor but also with regards to cells that make up the tumor microenvironment (TME) will be critical to our understanding of the process of tumorigenesis. In this review, we will discuss the role of ROS in tumorigenesis. We will also focus a significant portion of the review on describing how ROS regulate the biological processes of cells within the TME including cancer-associated fibroblasts (CAFs) and tumor-infiltrating immune (TII) cells. 

## 2. Role of Reactive Oxygen Species (ROS) in Tumorigenesis

The majority of endogenous ROS produced in cells result from metabolic reactions occurring within the mitochondria or peroxisome. However, there is a subset of ROS that are also produced by nicotinamide adenine dinucleotide phosphate (NAPDH) oxidases (NOX) which are a family of transmembrane proteins that transport electrons across biological membranes and catalyze the conversion of oxygen into superoxide. Superoxide is then further reduced by superoxide dismutases (SODs) to produce H_2_O_2_. ROS can also be produced from cyclooxygenases, lipoxygenases and thymidine phosphorylase [10].

ROS play an important role in tumorigenesis and affect multiple biological processes such as cell proliferation, genomic instability, inflammation, resistance to apoptosis and metabolic reprogramming. Increased levels of ROS are observed in a number of cancer cell lines [11]. In a tumor cell, ROS are primarily generated by the mitochondria. Mitochondria produce superoxide (O_2_•) from one-electron reduction of oxygen through the mitochondrial electron transport chain (ETC) [12]. Within the mitochondria, ROS are generated at a number of different sites, the most important being complexes I, II and III [13,14]. Complex I and II generate O_2_• in the mitochondrial matrix while complex III produces O_2_• in both the matrix and intermembrane space [15,16]. O_2_• generated in the mitochondrial matrix is converted to H_2_O_2_ by superoxide dismutase protein 2 (SOD2) [17]. Complex III-generated intermembrane space O_2_• can travel to the outer mitochondrial membrane and into the cytosol where it is converted into H_2_O_2_ by superoxide dismutase protein 1 (SOD1) [18]. Given access to the cytosol, it is thought that complex III-generated ROS are responsible for affecting cellular signaling [19].

As mentioned previously, ROS levels are often elevated in cancer, however, high levels of ROS can have deleterious effects therefore, cells have evolved mechanisms in order to maintain a proper balance of ROS. These mechanisms include peroxide scavenging systems (peroxidases) which control H_2_O_2_ levels by reducing H_2_O_2_ to H_2_O [20]. Production of mitochondrial ROS are also regulated by the availability of O_2_, the rate of electron flux through the ETC, the concentration of given electron carriers and the mitochondrial membrane potential [12,21]. Finally, the localization of mitochondria within the cell has an important effect on influencing cell signaling pathways as clustering of mitochondria to discrete areas of a cell can preferentially affect adjacent signaling pathways [22,23]. Together these mechanisms allow for balance of the effects of ROS which can be exploited by tumor cells in order to drive cells preferentially towards a proliferative state.

Mitochondrial ROS can stimulate multiple signaling pathways. Perhaps the most well-known is the requirement of mitochondrial ROS for the stabilization of hypoxia-inducible transcription factors (HIFs) under hypoxia [24,25,26,27]. HIF stabilization leads to initiation of a broad transcriptional program including regulation of genes important for angiogenesis [28]. In order for angiogenesis to occur proliferation of endothelial cells is required [29]. To that end, HIF upregulates the expression of vascular endothelial growth factor (VEGF). VEGF is a soluble growth factor that binds to VEGF receptors and activates signaling pathways important to endothelial cell proliferation [30]. The mitogenic effects of VEGF are mediated most commonly through the activation of the extracellular-signal-regulated kinase/mitogen-activated protein kinase (ERK/MAPK) pathway which is a potent stimulator of cell proliferation [31]. Mitochondrial ROS are also critical in the activation of T-cells [32]. Reduced levels of complex III-generated mitochondrial ROS in mice leads to the inability for sustained T-cell activation despite stimulation with CD3 or CD28 [32]. Furthermore, a study demonstrated that mitochondria translocate to the immunological synapse in a T-cell line, and mitochondrial H_2_O_2_ is required for T-cell receptor (TCR) signal transduction through MAPK signaling [33]. Together, this data suggests that mitochondrial ROS augment TCR signal transduction after antigen stimulation required for T-cell stimulation and proliferation. Together, mitochondrial ROS play an important role in stimulating physiological cell proliferation and can be exploited by a tumor to promote survival and growth.

**Figure 1:**
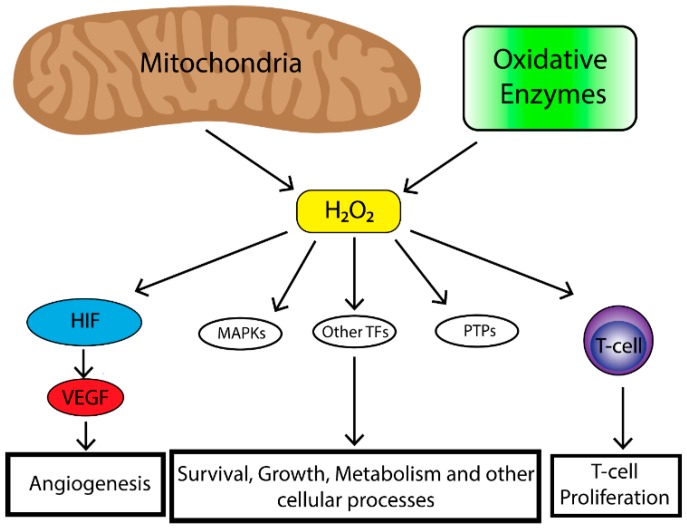
The mitochondria are the major contributor to cellular reactive oxygen species (ROS) levels while oxidative enzymes (e.g., NAPDH oxidases, cyclooxygenases, lipooxygenases and thymidine phosphorylase) also contribute to cellular ROS pooles. Mitochondrial ROS have many effects on cellular biology including, Mitogen-activated protein kinase (MAPK) (e.g., extracellular-signal-regulated kinase (ERK), p38 MAPK, Jun N-terminal kinase (JNK)), induction of transcription factors (e.g., nuclear factor kappa-light-chain-enhancer of activated B cells (NF-κβ), hypoxia-inducible transcription factors (HIF), activator protein 1 (AP-1), nuclear respiratory factor (NRF), heat shock factor 1 (HSF-1)) and deregulation of protein phosphatases (e.g., phosphatase and tensin homolog (PTEN)). This leads to enhancement of angiogenesis in the case of HIF, survival, growth, altered metabolism and other cellular processes through MAPKs, transcriptional factors and protein phosphatase and immune cell function and regulation.

Tumors produce high levels of ROS [11]. Initially it was felt that high levels of ROS contributed to tumorigenesis by oxidative damage to DNA leading to genomic instability [34]. However, studies also demonstrated increased protein expression of cellular antioxidants in cancer cells [35]. Thus, cancer cells have the ability to maintain elevated mitogenic signaling without incurring substantial oxidative damage. Indeed, oncogenes and/or tumor suppressor loss in cancer cells lead to ROS production. For example, a study in which oncogenic H-RasG12V was overexpressed in 3T3 fibroblasts demonstrated increased production of ROS required for mitogenic signaling [36]. Furthermore, mouse embryonic fibroblasts transformed by the loss of p53 tumor suppressor as well as expression of oncogenes Akt, H-RasG12V or KrasG12D demonstrated that mitochondrial ROS are required for anchorage-independent growth in soft agar [6]. Mitochondrial DNA mutations in several genes important for the function of the ETC are present in a number of human cancers [37]. These mutations also lead to increased levels of mitochondrial ROS production [38,39,40]. Loss of mitochondrial transcription factor A (TFAM) in a mouse model of K-ras driven lung cancer demonstrated reduced tumor growth [6]. TFAM is necessary for mitochondrial DNA replication. When TFAM is absent oxidative phosphorylation is impaired and hence levels of mitochondrial ROS are decreased [6]. Furthermore, this study demonstrated that mitochondrial ROS are required for anchorage independent growth in numerous cancer cell types [6] Taken together, the production of ROS by tumor cells plays an important role in driving tumorigenesis however, ROS production by other non-tumor infiltrating cells as well as the overall oxidative state of the local TME has profound effects on tumor biology (Figure 1).

## 3. Cancer-Associated Fibroblasts, ROS and the Tumor Microenvironment (TME)

The TME includes not only tumor cells but tumor lymphatics, tumor vessels, extracellular matrix, non-cancer stromal cells as well as chemical modulators (i.e., chemokines, cytokines, growth factors) and microbial populations. The extracellular matrix (ECM) and stroma include interstitial matrix as well as the basement membrane, and can act as a storage site for many growth factors and chemokines that can stimulate tumorigenesis. Non-cancer stromal cells include endothelial cells, pericytes, immune cells, activated adipocytes, mesenchymal stem cells (MSCs), normal fibroblasts and CAFs. Normal fibroblasts are responsible for ECM turnover and tissue homeostasis. They are fundamental in the processes of wound healing and senescence. Unlike normal fibroblasts, CAFs can be found at the margins of tumors or infiltrating into a tumor. Activated fibroblasts that are found in association with cancer cells are known as CAFs and play key roles in cancer initiation, progression and metastasis [41,42]. CAFs are further subdivided into fibroblasts and myofibroblasts. Alpha-smooth muscle actin (α-SMA)-positive myofibroblasts are noted to be the major population of CAFs present in tumors [43].

The major role of CAFs is to augment tumorigenesis [44]. Infiltrating CAFs are more proliferative than normal fibroblasts and activate specific signaling pathways important for the promotion of tumor growth [45,46,47]. CAFs residing at the margins of tumors but not within are characterized by their ability to promote cancer progression in vivo [48]. These CAFs are known to secrete factors such as CXCL12 which can go on to activate pro-tumorigenic pathways such as AKT in adjacent epithelial cells [49]. CAFs are present in almost all solid tumors. In certain tumors such as breast, pancreatic and prostate, CAFs can account for up to 80% of the tumor mass as they are responsible for the excessive growth of fibrous or connective tissue (desmoplasia) [50]. A high percentage of CAFs within cancer tissues is associated with poorer prognosis, increased infiltration of tumor-associated macrophages and epithelial to mesenchymal transition (EMT) [50].

**Figure 2:**
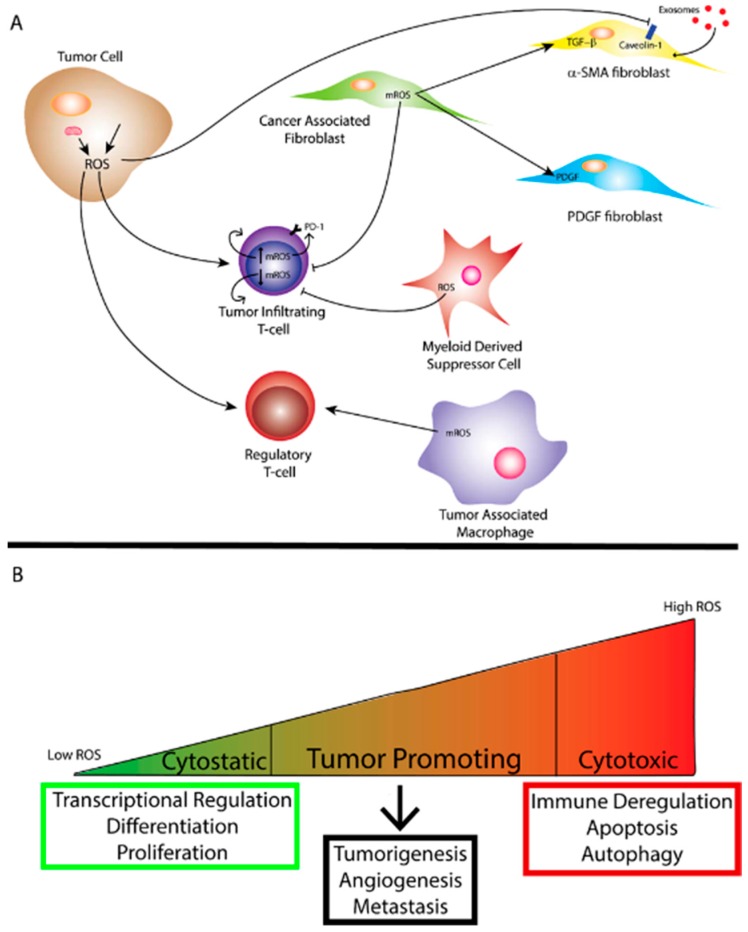
(**A**) Reactive oxygen species (ROS) generated by the mitochondria and/or exogenous sources within a tumor cell affect tumor immunity to promote a more tumorigenic environment. Mitochondrial ROS (mROS) can stimulate differentiation of cancer-associated fibroblasts (CAFs) and ROS produced by the tumor cell can facilitate uptake of exosomes through caveolin-1 inhibition leading to metabolic reprogramming of certain CAFs. ROS can also affect the function of tumor-infiltrating T-cells depending on the level of mROS. Myeloid-derived suppressor cells (MDSCs) and tumor-associated Macrophages (TAMs) also produce ROS that can affect the function of other immune cells and ROS can affect regulatory T-cell function as well. (**B**) The amount of ROS corresponds to differing effects on biological function. While cytostatic levels of ROS lead to maintenance of biological processes, cytotoxic levels of ROS lead to cell death as well as immune deregulation. Tumor promotion through ROS occurs when ROS reach super-physiological or cytostatic levels while avoiding levels conducive to cell death. As mentioned previously, oxidative stress can arise from tumor cells.

Desmoplasia is a marker of tumor progression and generates mechanical forces which can limit the lymphatic and blood supply to a tumor through compression of vessels in turn creating a hypoxic environment [51]. Furthermore, these mechanical forces can cause conversion of fibroblasts to myofibroblasts [51]. As previously mentioned, hypoxia stimulates the production of mitochondrial ROS and cancer cells produce higher levels of ROS than normal tissues which can influence CAF function [9,52]. CAFs can derive from epithelial, endothelial, hematopoietic stem cells, pericytes or adipocytes as well as resident fibroblasts present in stromal tissue [53,54,55,56,57,58,59]. A large proportion of CAFs identified in aggressive adenocarcinomas express smooth-muscle α-actin (α-SMA) and, therefore, are called myofibroblasts [60]. Myofibroblasts’ major function is in wound healing and repair and in tumors these cells can act as drivers for deranged chronic wound healing. Several studies have demonstrated that ROS can be a driver for myofibroblast differentiation. Several studies reported the importance of ROS in the fibroblast to myofibroblast transition (Figure 2A). Transforming growth factor beta 1 (TGF-β1) as well stromal cell-derived factor 1 (SDF-1) and others play a major role in driving the transition from fibroblast to myofibroblast. It is well known that mitochondrial-ROS are required for TGF-β1 activation. Indeed, when fibroblasts were exposed to a pharmacologic inhibitor of mitochondrial-ROS, TGF-β1 expression levels were reduced [61]. Fibroblast to myofibroblast conversion can also be induced with SDF-1 in an ROS-dependent manner [58,60]. Furthermore, fibroblasts exposed to chronic oxidative stress can also differentiate into myofibroblasts [9,60]. Fibroblasts isolated from mouse models of oxidative stress in which key antioxidant transcription factors were depleted demonstrated a conversion to myofibroblasts which could be reversed with the long-term treatment with exogenous antioxidants [60,62]. Additionally, decreased ROS levels due to upregulation of antioxidant enzymes such as glutathione peroxidase 3 and thioredoxin reductase I within fibroblasts from prostate cancer inhibits differentiation into myofibroblasts [63]. Taken together these observations demonstrate that ROS can promote myofibroblast differentiation in human tumors.

ROS can also affect proliferation and migration of CAFs. As discussed previously, CAFs exist as a heterogeneous population with different populations expressing certain markers differentially. While α-SMA myofibroblasts represent the majority of CAFs there are other markers that have been used to detect subtypes of fibroblasts [9]. However, it is unclear whether these subtypes truly represent distinct sub-populations of fibroblasts. Studies have suggested that ROS can play a role in impacting fibroblast subtype [9]. One subtype of fibroblast that may be affected by ROS are platelet-derived growth factor beta (PDGF-β) fibroblasts. ROS play an important role in PDGF signaling through the inhibition of phosphatases [64,65,66,67]. The activation of PDGF signaling stimulates fibroblast growth and motility. Interestingly, a study demonstrated that upon PDGF stimulation of normal human fibroblasts, NOX4 and DUOX4, two enzymes responsible for increasing levels of ROS within cells, modulate cell cycle entry [68]. Together these studies indicate that in PDGF-β fibroblasts ROS could play an integral role in affecting fibroblast proliferation and migration. Another fibroblast marker that could potentially be affected by ROS is Caveolin-1 (CAV-1). Studies demonstrate that when fibroblasts and tumor epithelial cells are co-cultured in the presence of oxidative stress, CAV-1 is degraded in fibroblasts which can be prevented by the treatment of antioxidant and autophagy inhibitors [69,70,71]. Taken together, these studies suggest that ROS produced by fibroblasts play an important role in CAF activation and differentiation. However, ROS and other metabolic byproducts are also produced in large quantities by tumor cells and could also play a role in CAF function. ROS produced by CAFs, as mentioned previously, could also augment tumorigenesis. 

H_2_O_2_ is produced from tumor epithelial cells and can diffuse into other tissues and cells. H_2_O_2_ is also implicated in intracellular signaling pathway activation. Certain studies focused on understanding how H_2_O_2_ affects the tumor microenvironment (TME) and stroma. In these studies, breast cancer cells were co-cultured with CAFs to demonstrate the effect of tumor-generated H_2_O_2_ on CAFs. Interestingly, tumor H_2_O_2_ led to a reduction in mitochondrial function, increase in glucose uptake and increase ROS in CAFs [70,72]. Furthermore, co-cultured cancer cells demonstrated increased mitochondrial activity and decreased GLUT1 expression along with decreased glucose uptake [70]. This cross-talk between tumor cells and CAFs could be abrogated with the addition of catalase [70]. Finally, fibroblasts co-cultured with breast cancer cells displayed Caveolin-1 (CAV-1) downregulation and increased expression of markers for myofibroblasts [70]. This suggests that tumor cells produce ROS which can directly reprogram CAFs to potentially create a more pro-tumorigenic microenvironment.

Caveolins, such as CAV-1, are unique proteins which are found on multiple cell types and help to form caveolae which are plasma membrane invaginations. CAV-1 expression is mediated by self-digestion or autophagy [73,74]. Human CAFs will usually display reduced CAV-1 expression as compared to normal fibroblasts. Reduced CAV-1 expression is associated with increased glycolysis and reduced mitochondrial function and this decrease in CAV-1 expression is thought to be mediated by tumor cell oxidative stress induced autophagy [8,74,75]. Fibroblast-mediated degradation of CAV1 can be abrogated with antioxidants and autophagy inhibitors [70,71]. CAV-1 expression has not only been implicated in the induction of a metabolic switch but also in autophagy/mitophagy activity and remodeling of the microenvironment [9]. Interestingly, CAV-1 expression in lung cancer cells is differentially affected by different types of ROS. For example, hydroxyl radical up-regulates CAV-1 while O_2_• and H_2_O_2_ down-regulated CAV-1 expression. It should also be noted that degradation of CAV-1 leads to increased exosomal uptake into cells [76].

Exosomes are a subtype of extracellular vesicles (EVs) deriving from intraluminal endosomal vesicles. Exosomes are made up of a lipid bilayer and contain proteins, mRNAs, lipids, miRNAs and free metabolites which are released into the cytosol of target cells after internalization [77]. Cancer-derived EVs are able to transform non-malignant cells in the tumor microenvironment in order to promote tumorigenesis [78,79]. EVs, therefore, provide a mechanism for cellular crosstalk. Indeed, studies show that exosomes have the ability to reprogram recipient cells and are able to modulate proliferation, survival and immune effector status in recipient cells [80]. More recently, Zhao et al., demonstrated that exosomes isolated from prostate and pancreatic cancer patient-derived CAFs can inhibit mitochondrial oxidative phosphorylation in cancer cells increasing glycolysis and reductive carboxylation [81]. 

Exosomes are taken up into cells through different pathways and the process by which exosomes are taken up into cells is controversial. Recently, a study demonstrated that exosomes derived from glioblastoma (GBM) cells are internalized through non-classical, lipid-raft dependent endocytosis [76]. The authors then demonstrate that the lipid raft associated protein, CAV-1, negatively regulates the uptake of exosomes [76]. Previously, it was mentioned that tumor cells induce oxidative stress which leads to the autophagic degradation of CAV-1 [8,74]. Together, these studies suggest a pathway by which tumor cells produce ROS which signal to fibroblasts and lead to the degradation of CAV-1 and, therefore, increased exosomal uptake. The effect of increased exosomal uptake could then lead to increased influx of metabolites and metabolic reprogramming of the fibroblast to a more pro-tumorigenic CAF (i.e., myofibroblast) (Figure 2A). Indeed, Zhao et al. demonstrated that exosomes isolated from pancreatic and prostate cancer CAFs contained high amounts of glutamine, lactate and acetate as well as many other amino acids and metabolites suggesting a role for exosomes in anapleurosis and lipogenesis [81]. 

Autophagy is a pathway by which cytoplasmic organelles or components are sequestered into an autophagosome and delivered to lysosomes for degradation. Autophagy is essential for survival, differentiation, development and metabolism and is involved in many disease states, such as cancer. Autophagy can be stimulated by cellular stress including ROS [82,83]. ROS are able to regulate autophagy both directly and indirectly [84,85,86]. ROS-induced autophagy has been demonstrated to protect against oxidative damage suggesting an ROS-dependent negative feedback loop to regulate oxidative stress within cells [87]. Defective autophagy is observed in multiple tumors which supports a tumor suppressive role [88,89,90]. However, studies also show that autophagy has tumor-promoting functions which implies autophagy function is context dependent in cancer [91,92]. Aside from cancer type, this context-dependent functioning likely applies to cells present in the TME as well. There is now evidence that ROS can provide cross-talk between CAFs and tumor cells through autophagy that can create a more pro-tumorigenic environment. A previous study performed in a xenograft model of breast cancer demonstrated that HIF-1α-dependent activation of autophagy in stromal cells enhances tumorigenicity [93]. Given the effect of mitochondrial ROS on HIF-1α, it can be surmised that ROS in stromal cells may modulate tumorigenicity of cancer cells through induction of autophagy. Another study explored CAFs isolated from ovarian cancer tissues as well as normal fibroblasts from benign tissue and found that CAFs are resistant to oxidative stress and this process is mediated through autophagy [94]. CAFs could act as central mediators of oxidative stress within the TME and help to give tumor cells the ability to circumvent the cytotoxic effects of elevated TME ROS levels. Tumor cells can also affect the cells of TME through autophagy and mitophagy, which is the selective degradation of mitochondria by autophagy. Several studies demonstrated that tumor cells can induce increased metabolism in CAFs which also induces autophagy and mitophagy allowing for the recycling of important biomolecules and metabolic precursors [95,96]. It would be expected that the byproduct of this would also be generation of ROS in CAFs which could also help reprogram them to a more pro-tumorigenic fibroblast.

## 4. Tumor Immunity and ROS

As discussed previously, the TME compromises a large number of different cell types including tumor-infiltrating leukocytes (TILs). TILs include myeloid-derived suppressor cells (MDSCs), tumor-associated macrophages (TAMs) and regulatory T cells (Tregs) as well as other immune cells. MDSCs, TAMs and Tregs work concomitantly to suppress the immune response to a tumor. Studies show that an immunosuppressive microenvironment allows for greater tumor invasion, metastasis and resistance to treatments [97]. While ROS are known to be promoters of tumor progression one way in which they may achieve this is through immune cell suppression (Figure 2A). In this section we will focus mainly on ROS effects on tumor-infiltrating T-cells. T-cells are essential to the host immune response to cancer. Tumor-infiltrating cytotoxic T-cells (i.e., CD8^+^ T-cells) play a pivotal role in the anti-tumor immune response. However, during cancer progression the tumor microenvironment becomes immunosuppressive and T-cell cytotoxicity is inhibited. As mentioned previously, ROS are also associated with a more immunosuppressive tumor microenvironment. ROS have been implicated in a variety of roles with regards to the activation and regulation of T-cells in the microenvironment. High levels of ROS in the TME inhibit T-cell proliferation and anti-tumor function. Furthermore, ROS produced by other cells within the tumor microenvironment lead to T-cell hyporesponsiveness in cancer patients [98]. Alternatively, a low level of ROS is required for T-cell activation, proliferation and function [99,100]. T-cell activation occurs through stimulation of the TCR and co-stimulatory receptors which induce signaling pathways and transcription factors. Furthermore, TCR-dependent calcium influx into CD4^+^ T-cells leads to the generation of mitochondrial ROS which are required for CD4^+^ T-cell activation [32,101]. Both complex I and III of the ETC have been implicated as sources of mitochondrial ROS essential for T-cell activation [32,102]. Tumor infiltrating T-cells display loss of mitochondrial function which can be rescued by expression of PGC1α, a key player in mitochondrial biogenesis, and subsequently restores antitumor activity [103]. Furthermore, a more recent study demonstrated that in clear cell renal cell carcinoma tumors CD8 TILs were present but they were functionally and metabolically impaired [104]. They were noted to generate large amounts of mitochondrial ROS leading to downregulation of mitochondrial superoxide dismutase 2 (SOD2) [104]. This effect could be rescued with MitoQ as well as MitoTEMPO, both mitochondrial ROS scavengers, as evidenced by an increase in CD8 TIL activation [104]. Another study demonstrated that T cells modified with a bicistronic expression vector chimeric Ag receptor (CAR) co-expressing catalase (CAT) exhibited reduced intracellular oxidative stress which resulted in the increased ability of CAR-CAT T-cells to lyse tumor cells in an antigen-specific manner [105]. Interestingly, these CAR-CAT T-cells were able to function under extracellular oxidative stress unlike traditional CAR T-cells [105]. CAR T cells rely on the genetic transfer of tumor-specific TCRs and CARs into T cells from peripheral blood. Unfortunately, in solid tumors CAR T-cells not only have to reach their targets but must also survive and function within the unfavorable tumor microenvironment. The above study highlights the importance of understanding ROS biology in order to affect biological consequences. 

PD-1 is a surface receptor expressed mostly by activated T-cells and acts as a negative regulator of the immune response. PD-1 binds to one of its two ligands (PD-L1 or PD-L2) which leads to phosphorylation of its receptor and recruitment of Src homology region 2 domain-containing phosphatase-2 (SHP-2) which leads to dephosphorylation of TCR activation molecules [106,107]. This leads to the “unmasking” of the tumor and activation of tumor-reactive cytotoxic T-lymphocytes (T-CTLs). How PD-1 blockade results in abrogation of tumor tolerance remains largely unknown. However, recently a paper was published suggesting a role for ROS and mitochondrial activation in PD-1 blockade induction of T cell-dependent antitumor activity [108]. In this study, the authors found that T-CTLs isolated from mice treated with PD-L1 blockade have increased mitochondrial mass, membrane potential, superoxide and cellular ROS indicating these cells have higher rates of mitochondrial metabolism [108]. Furthermore, the authors found that treatment of these cells with a ROS generator (tert-butyl hydroperoxide) or a mitochondrial respiratory chain uncoupler (FCCP) further synergized the effect of PD-1 blockade on tumor growth inhibition [108]. This suggests that modulating mitochondrial activity and mitochondrial ROS in immune cells such as T-CTLs could have profound effects on response to PD-1 blockade. Perhaps non-responders to PD-1 inhibitors have lower levels of mitochondrial activation and hence ROS, which allows for suboptimal activation of T-CTLs.

Another study demonstrated that sensitivity to PD-1 blockade in mouse tumor cell lines depended on the cells’ ability to consume oxygen and hypoxia [109]. The authors found that when they treated cells with metformin oxygen consumption was inhibited in vitro and in vivo which resulted in intratumoral hypoxia [109]. When mice were treated with the combination of metformin and PD-1 blockade improved intratumoral T-cell function and tumor clearance was observed [109]. This suggests that lower levels of ROS and, therefore, a less hypoxic tumor environment allows for increased efficacy of PD-1 blockade immunotherapy. It is conceivable then that lower levels of global ROS and hypoxia in the tumor microenvironment coupled with increased intracellular mitochondrial ROS in specific tumor-infiltrating cells may produce the most efficacious response to PD-1 blockade.

The dichotomy of the studies described highlights the importance of understanding how mitochondrial ROS can be modulated to different levels to affect biological outcomes (Figure 2A). The yin and yang of ROS is potentially a therapeutic target within the realm of immuno-oncology, as different levels of ROS capitulate profoundly different immunologic programs (Figure 2B). More recently, immunotherapy targeting PD-1/PD-L1 blockade has changed the landscape of cancer treatment and significantly increased the survival rate in cancer patients. Currently, this treatment is approved for patients with melanoma, non-small cell lung cancer (NSCLC), kidney cancer, Hodgkin lymphoma and head and neck cancer. However, it should be noted that while durable responses are significant in many patients treated with PD-1/PD-L1 blockade approximately 30–50% of patients remain unresponsive or less responsive to PD-1/PD-L1 blockade [110,111,112]. Understanding how to improve response to PD-1/PD-L1 blockade could have a significant effect on survival in patients. 

Regulatory T-cells (Tregs) are also present in the TME and provide another source of immunosuppression, decreasing tumor reactive cytotoxic T-cell immunity. Studies suggest that the balance between immunosuppressive Tregs and cytotoxic T-cells may be metabolically regulated [113,114,115,116]. As discussed, ROS and oxidative stress in the tumor microenvironment help drive tumor immunity through effects on tumor-infiltrating immune cells. A recent paper showed that increased ROS and oxidative stress in the tumor environment led to more potent immunosuppression by Tregs [117]. The authors found that apoptotic Tregs were actually more efficient at suppressing T-cell activation in vitro and in vivo [117]. Furthermore, these apoptotic Tregs abolished the therapeutic efficacy of PD-L1 blockade in tumor-bearing mouse models [117]. This was thought to be mediated through the release of large amounts of ATP and adenosine which are immunosuppressive [117]. This raises the therapeutic possibility of targeting ROS in the tumor microenvironment in order to reduce ROS levels in turn inhibiting the immunosuppressive activity of Tregs. On this point, a recent study demonstrated that reduced levels of mitochondrial ROS led to decreased Treg differentiation [118]. Kunisada et al. demonstrated that metformin reduced the number of tumor-infiltrating Tregs by inhibiting differentiation of naïve CD4^+^ T-cells into Tregs through forkhead box P3 (Foxp3) protein [118]. Furthermore, the authors demonstrated that metformin induced metabolic reprogramming of Tregs to a more glycolytic state [118]. Metformin is a complex I inhibitor and thus reduces the pool of Complex I generated mitochondrial ROS. It is likely that the decrease in mitochondrial ROS in metformin treated T-regs also has an effect on altering transcriptional programs important for metabolic reprogramming. Additionally, a recent study by Weinberg et al. found that complex III of the mitochondrial ETC is required for Treg suppressive activity [119]. Through mouse experiments, the authors demonstrate that the loss of Rieske iron-sulfur protein (RISP), an essential subunit of mitochondrial complex III, led to loss of Treg suppressive function. ROS generated at complex III are important signaling transducers [3,120]. Specific mitochondrial targeted antioxidants exist [6,121] and it would be interesting to see if those drugs mirror the effect of RISP knock out on Tregs. Based on the above studies, it is possible that by decreasing the levels of mitochondrial ROS with metformin or mitochondrial targeted antioxidants, Tregs become less immunosuppressive, allowing for increased cytotoxic T-cell tumoricidal effect. 

Taken together, this again suggests that the level of ROS within a specific cell type has important consequences for the function of that cell. As discussed, high levels of ROS in CTLs may have an anti-tumoricidal effect while low levels of ROS in Tregs seem to be associated with decreased immunosuppression. Furthermore, similar levels of ROS may also have contradicting effects in varying cell types. As shown above, while in CD8 TILs from renal cell carcinoma, high levels of ROS led to impairment and lack of anti-tumor response, high levels of ROS in TILs from mice with colon carcinoma treated with anti-PD-1 blockade were associated with increased tumoricidal effects. Greater research into how ROS within tumor infiltrating immune cells as well as extracellular ROS involvement in modulating tumor immunity will be needed to further characterize how differing levels, locations and types of ROS are affecting tumor immunity. 

There is also evidence that other TILs such as MDSC and TAMs are regulated by ROS. MDSCs are one of the major immunosuppressive cell types within the tumor microenvironment [122]. These cells induce immunosuppression through the inhibition of T-cells. More recently, a study demonstrated that tumor-induced MDSCs could suppress T cell proliferation helping to promote colorectal cancer cell growth through the production of ROS [123]. In another study, MDSC’s immunosuppressive effects on T-cells could be completely abrogated with the use of ROS inhibitors [124]. It should be noted that ROS suppress T-cell immune responses by inhibiting recognition between the T-cell receptor (TCR) and the MHC-peptide complex. This was highlighted in a study which demonstrated that co-culturing MDSCs with T-cells in the presence of catalase, a ROS inhibitor, impaired MDSC-mediated T-cell proliferation [125]. Finally, it is important to understand that while high levels of ROS are immunosuppressive, low levels of ROS are likely important for T-cell activation [126]. TAMs are another immunologic class of cells that are present in the tumor microenvironment. TAMs are thought to be critical mediators of inflammation and tumorigenesis. ROS are implicated in macrophage activation and signaling. Furthermore, a study demonstrated that macrophage-derived ROS induce Tregs [127]. This suggests that ROS derived from macrophages can have an immunosuppressive effect. Another study demonstrated that ROS are required to promote a more invasive phenotype in TAMs isolated from melanomas and this effect was mediated through ROS-dependent tumor necrosis factor α secretion [128]. It is also important to note that the authors of this study found that TAMs from melanomas expressed elevations in multiple mitochondrial biogenesis and respiratory chain genes indicating mitochondrial ROS as one of the main sources of oxidative stress within TAMs [128]. These studies highlight the role of ROS not only as inducers of oxidative stress but also as mediators of immune regulation within the tumor microenvironment, important in promoting tumorigenesis. 

## 5. The Microbiome and ROS

Another element that comprises the TME is the microbiome. Over the past few years much research has revolved around the relationship between host microbiota and how the disruption in homeostasis of microbial communities (dysbiosis) affects pathologic conditions such as cancer. It is well known that host microbiota can promote carcinogenesis through induction of pro-inflammatory toxins, alterations in signaling pathways or through impairment of antitumor immune functions [129,130,131,132]. Another way host microbiota can potentially induce a tumorigenic state is through production of ROS [133]. For example, *Enterococcus faecalis*, a commensal strain of bacteria, can produce large amounts of extracellular superoxide that is converted to H_2_O_2_ which can damage eukaryotic cell DNA [134]. Pathogenic *Bacteroides fragilis* produces its toxin, which upregulates bacterial polyamine catabolism pathways, generating ROS that can cause DNA damage and lead to tumor formation in the colon [135]. Further studies have demonstrated that certain species of bacteria utilize bile acids for their respiration producing DNA-damaging ROS by-products that can induce gastrointestinal cancers [136,137]. Alternatively, injured mucosa relies upon redox signaling and ROS for repair. Microbiota produce and excrete formylated peptides which activate colonic epithelial formyl peptide receptors inducing localized ROS generation that activates signaling pathways important for epithelial wound healing [138]. Taken together this illustrates the dichotomous role of ROS in the microenvironment: as damaging agents as well as growth and healing promoters. In order to understand how ROS fully affect the TME, more research will need to be undertaken to explore the specific role bacterial species-specific ROS have upon tumor, immune and stromal cell function. 

While much data supports a role for microbiota derived ROS in tumorigenesis within the TME, studies have associated changes in host mitochondrial metabolism with changes in host microbiota [139]. A recent study by Yardeni et al. demonstrated that host mitochondria influence gut microbiome diversity through ROS [140]. By examining the gut microbiota of mice with various mutations in genes that alter mitochondrial function the authors were able to show that mitochondrial genetic variations altered the composition of the gut microbiota. Further analysis of mitochondrial DNA variants associated with an altered gut microbiome suggested that diversity correlated with host ROS production. Furthermore, they were able to demonstrate that modulation of ROS levels within mice led to altered gut microbiota. The authors find that decreased mitochondrial ROS leads to a higher diversity of species within the gut microbiota. Recently, a study demonstrated melanoma patients who respond to immunotherapy, have increased gut microbiota diversity [141]. Taken together, this suggests that modulation of mitochondrial ROS could be used to enhance a cancer patient’s sensitivity to immunotherapy.

## 6. Therapeutic Implications of ROS Targeting

As reviewed, ROS lies at a crossroads potentially linking the tumor and immune microenvironment. Therefore, ROS are attractive therapeutic targets as a modality to manipulate the tumor and microenvironment cross-talk and improve cancer outcomes. Recently, targeting of mitochondrial complex I, important for mitochondrial ROS generation, has gained clinical traction. Over 10 years ago, metformin, widely used in the treatment of type II diabetes mellitus, demonstrated reduced risk of cancer in diabetic patients [142]. Further studies, exploring metformin’s mechanism of action demonstrated the ability of metformin to inhibit complex I in vitro [143,144,145]. A further study demonstrated that targeting mitochondrial complex I with metformin led to inhibition of complex I, reduction of complex I generated ROS and reduced tumorigenesis in xenograft mouse models [146]. Impart, this led to a multitude of clinical studies which to date have produced disappointing results. Currently, there are multiple clinical trials in progress or actively recruiting centered around the use of metformin in the treatment of cancer. These new trials largely involve use of metformin in combination with immunotherapy (NCT03311308, NCT03874000, NCT03994744), chemotherapy (NCT02122185, NCT01310231, NCT03238495, NCT02122185, NCT03243851) doxycycline (NCT02874430), and intermittent fasting (NCT03709147) in specific sub-populations of cancer patients. There is also significant evidence to show that metformin may influence tumor progression by modulating the TME [147]. Through regulation of complex I ROS and mitochondrial metabolism, metformin has the ability to skew the phenotype of TME cell populations. Further studies could potentially address combining metformin with specific cytokine inhibitors, such as IL-6, IL-17, or FOXP3 along with PD-1/PD-L1 to more effectively remodel the TME. Of note, metformin is a weak complex I inhibitor and other clinical studies are currently underway using stronger, more specific mitochondrial ROS inhibitors. In a Phase I study, ME-344, an isoflavone-derived complex I mitochondrial inhibitor, is being evaluated in early stage HER2-negative breast cancer patients in combination with bevacizumab, an anti-vascular endothelial growth factors A (VEGF-A) inhibitor, assessing whether combination of ME-344 and bevacizumab can offset the metabolic changes that occur with anti-VEGF treatment (NCT02806817). These metabolic changes with anti-angiogenic therapies are linked to drug resistance. It is well known that chemotherapy induces ROS generation and that over time tumors become resistant to chemotherapy. Using metformin in combination with second line treatments or at specific time-points during front-line treatment may potentially abrogate or prolong the development of drug resistance. Finally, other clinical studies are focused on finding targeted approaches to blocking not only complex I but complex III of the ETC as well, which is thought to be the major generator of mitochondrial ROS important for induction of cellular signaling pathways. One such study is being performed in patients with chronic myelogenous leukemia (CML) using the antibiotic, Tigecycline, which impairs mitochondrial DNA translation and subsequently inhibits formation of complex I, III, IV and V of the ETC (NCT02883036). In the future, understanding and repurposing drugs that have specific effects on mitochondrial ROS generation could potentially lead to combination therapies that improve outcomes in cancer patients. 

Another potential target for therapeutic intervention is the TCA cycle. Cancers have dysregulated metabolism and it is now well appreciated that mitochondrial metabolism is required for a tumor’s growth. Recent studies have focused on targeting specific TCA cycle enzymes. One such drug is CPI-613 which is a lipoate analog. Lipoate is a co-factor for both pyruvate dehydrogenase and the alpha-ketoglutarate-dehydrogenase complex both required for rate-limiting steps in the TCA cycle. Inhibition of these enzymes causes mitochondrial dysregulation. As expected, this impairment leads to induction of mitochondrial ROS [148]. This induction of mitochondrial ROS by CPI-613 has the potential to tip the redox balance within a tumor cell towards cytotoxicity (Figure 2B). CPI-613 would also be expected to have effects on immune cells present in the TME. For example, immunosuppressive immune cells such as M2 macrophages, Tregs and MDSCs depend on oxidative phosphorylation and mitochondrial metabolism. CPI-613 can potentially target these pro-tumor cell types and tip the redox balance in favor of cellular dysfunction or toxicity. Already there is exciting Phase I data in metastatic pancreatic cancer patients suggesting that CPI-613 in combination with standard of care chemotherapy significantly improves overall response rates and potentially progression free survival [149]. Currently, there are several clinical trials exploring CPI-613 in combination with chemotherapy in a number of different tumor subtypes (NCT02168140, NCT02232152, NCT03699319, NCT02484391). In the future, combining CPI-613 with immunotherapy could be a potential area of research as well as understanding how CPI-613 could be utilized in patients who have developed drug resistance.

Finally, another attractive therapeutic target is the antioxidant machinery. Recently, RTA-408, also known as omaveloxolone, has been studied in melanoma. RTA-408 is a semisynthetic triterpenoid known to induce nuclear factor erythroid 2-related factor 2 (Nrf2) which is a major cellular regulator of protection against oxidative or electrophilic stress [150,151,152]. Through this mechanism, RTA-408 suppresses reactive oxygen and nitrogen species which has been demonstrated in tumor xenografts [153]. This effect of RTA-408 has also been shown in MDSCs [153]. At higher concentrations RTA-408 also selectively inhibits tumor growth through nuclear factor kappa-B kinase subunit [153]. Together, this drug can potentially target tumor and the immunosuppressive TME concurrently. Currently, there is a clinical Phase I/II study underway combining RTA-408 with immunotherapy in patients with melanoma. 

Altogether, targeting ROS within the tumor and TME has the potential to improve outcomes to current treatments. While it is unlikely that targeting ROS alone will derive therapeutic benefit, as evidenced by single agent metformin trials, in combination with cytotoxic or immune-regulating agents the potential exists to significantly improve response and survival. Understanding how to modulate the sensitive redox balance within and between tumor and different TME-infiltrating immune cells will potentially lead to more efficacious treatment. In the future, cell-specific ROS modulators may hold the key to optimizing potential treatments utilizing ROS as a target. 

## 7. Conclusions

ROS play an important role in maintaining physiological homeostasis within healthy cells. In tumor cells, the exploitation of pathways that lead to ROS production which result in a pro-growth, pro-tumorigenic environment occurs. It is now known that not only tumor cells but the TME including non-cancer stromal cells such as CAFs as well as tumor-infiltrating immune cells play important roles in the cross-talk between tumor and environment in order to drive tumorigenesis. It is becoming increasingly clear that metabolic reprogramming and subsequent ROS generation are essential to this crosstalk. The differential effects of varying levels of ROS on biological outcomes within the cells that encompass a tumor and its microenvironment are becoming increasingly important to our understanding of tumor initiation, growth and progression. Understanding how to exploit ROS in order to modulate cell-specific biological functions will not only help with the possible creation of new anticancer treatments but may also allow for increased efficacy of those treatments already in existence. 

## References

[1] Rhee S.G. (2006). Cell signaling. H_2_O_2_ a necessary evil for cell signaling. Science.

[2] Diebold L., Chandel N.S. (2016). Mitochondrial ROS regulation of proliferating cells. Free Radic. Biol. Med..

[3] Weinberg F., Chandel N.S. (2009). Reactive oxygen species-dependent signaling regulates cancer. Cell Mol. Life Sci..

[4] Weinberg F., Chandel N.S. (2009). Mitochondrial metabolism and cancer. Ann. N. Y. Acad. Sci..

[5] Hanahan D., Weinberg R.A. (2011). Hallmarks of cancer: The next generation. Cell.

[6] Weinberg F., Hamanaka R., Wheaton W.W., Weinberg S., Joseph J., Lopez M., Kalyanaraman B., Mutlu G.M., Budinger G.R., Chandel N.S. (2010). Mitochondrial metabolism and ROS generation are essential for Kras-mediated tumorigenicity. Proc. Natl. Acad. Sci. USA.

[7] Kong H., Chandel N.S. (2018). Regulation of redox balance in cancer and T cells. J. Biol. Chem..

[8] Martinez-Outschoorn U.E., Balliet R.M., Rivadeneira D.B., Chiavarina B., Pavlides S., Wang C., Whitaker-Menezes D., Daumer K.M., Lin Z., Witkiewicz A.K. (2010). Oxidative stress in cancer associated fibroblasts drives tumor-stroma co-evolution: A new paradigm for understanding tumor metabolism, the field effect and genomic instability in cancer cells. Cell Cycle.

[9] Costa A., Scholer-Dahirel A., Mechta-Grigoriou F. (2014). The role of reactive oxygen species and metabolism on cancer cells and their microenvironment. Semin. Cancer Biol..

[10] Storz P. (2005). Reactive oxygen species in tumor progression. Front. Biosci..

[11] Szatrowski T.P., Nathan C.F. (1991). Production of large amounts of hydrogen peroxide by human tumor cells. Cancer Res..

[12] Murphy M.P. (2009). How mitochondria produce reactive oxygen species. Biochem. J..

[13] Quinlan C.L., Perevoshchikova I.V., Hey-Mogensen M., Orr A.L., Brand M.D. (2013). Sites of reactive oxygen species generation by mitochondria oxidizing different substrates. Redox Biol..

[14] Nickel A., Kohlhaas M., Maack C. (2014). Mitochondrial reactive oxygen species production and elimination. J. Mol. Cell. Cardiol..

[15] Turrens J.F. (2003). Mitochondrial formation of reactive oxygen species. J. Physiol..

[16] Muller F.L., Liu Y., Van Remmen H. (2004). Complex III releases superoxide to both sides of the inner mitochondrial membrane. J. Biol. Chem..

[17] Fridovich I. (1997). Superoxide anion radical (O_2_-), superoxide dismutases, and related matters. J. Biol. Chem..

[18] Han D., Antunes F., Canali R., Rettori D., Cadenas E. (2003). Voltage-dependent anion channels control the release of the superoxide anion from mitochondria to cytosol. J. Biol Chem..

[19] Orr A.L., Vargas L., Turk C.N., Baaten J.E., Matzen J.T., Dardov V.J., Attle S.J., Li J., Quackenbush D.C., Goncalves R.L. (2015). Suppressors of superoxide production from mitochondrial complex III. Nat. Chem. Biol..

[20] Winterbourn C.C. (2013). The biological chemistry of hydrogen peroxide. Methods Enzymol..

[21] Echtay K.S., Murphy M.P., Smith R.A., Talbot D.A., Brand M.D. (2002). Superoxide activates mitochondrial uncoupling protein 2 from the matrix side. Studies using targeted antioxidants. J. Biol. Chem..

[22] Frederick R.L., Shaw J.M. (2007). Moving mitochondria: Establishing distribution of an essential organelle. Traffic.

[23] Al-Mehdi A.B., Pastukh V.M., Swiger B.M., Reed D.J., Patel M.R., Bardwell G.C., Pastukh V.V., Alexeyev M.F., Gillespie M.N. (2012). Perinuclear mitochondrial clustering creates an oxidant-rich nuclear domain required for hypoxia-induced transcription. Sci. Signal.

[24] Wang G.L., Jiang B.H., Rue E.A., Semenza G.L. (1995). Hypoxia-inducible factor 1 is a basic-helix-loop-helix-PAS heterodimer regulated by cellular O_2_ tension. Proc. Natl. Acad. Sci. USA.

[25] Martinez-Reyes I., Diebold L.P., Kong H., Schieber M., Huang H., Hensley C.T., Mehta M.M., Wang T., Santos J.H., Woychik R. (2016). TCA Cycle and Mitochondrial Membrane Potential Are Necessary for Diverse Biological Functions. Mol. Cell.

[26] Chandel N.S., Maltepe E., Goldwasser E., Mathieu C.E., Simon M.C., Schumacker P.T. (1998). Mitochondrial reactive oxygen species trigger hypoxia-induced transcription. Proc. Natl. Acad. Sci. USA.

[27] Chandel N.S., McClintock D.S., Feliciano C.E., Wood T.M., Melendez J.A., Rodriguez A.M., Schumacker P.T. (2000). Reactive oxygen species generated at mitochondrial complex III stabilize hypoxia-inducible factor-1alpha during hypoxia: A mechanism of O_2_ sensing. J. Biol. Chem..

[28] Pugh C.W., Ratcliffe P.J. (2003). Regulation of angiogenesis by hypoxia: Role of the HIF system. Nat. Med..

[29] De Smet F., Segura I., De Bock K., Hohensinner P.J., Carmeliet P. (2009). Mechanisms of vessel branching: Filopodia on endothelial tip cells lead the way. Arter. Thromb. Vasc. Biol..

[30] Ferrara N. (2009). VEGF-A: A critical regulator of blood vessel growth. Eur. Cytokine Netw..

[31] Hoeben A., Landuyt B., Highley M.S., Wildiers H., Van Oosterom A.T., De Bruijn E.A. (2004). Vascular endothelial growth factor and angiogenesis. Pharmacol. Rev..

[32] Sena L.A., Li S., Jairaman A., Prakriya M., Ezponda T., Hildeman D.A., Wang C.R., Schumacker P.T., Licht J.D., Perlman H. (2013). Mitochondria are required for antigen-specific T cell activation through reactive oxygen species signaling. Immunity.

[33] Gill T., Levine A.D. (2013). Mitochondria-derived hydrogen peroxide selectively enhances T cell receptor-initiated signal transduction. J. Biol. Chem..

[34] Ames B.N., Shigenaga M.K., Hagen T.M. (1993). Oxidants, antioxidants, and the degenerative diseases of aging. Proc. Natl. Acad. Sci. USA.

[35] Schafer Z.T., Grassian A.R., Song L., Jiang Z., Gerhart-Hines Z., Irie H.Y., Gao S., Puigserver P., Brugge J.S. (2009). Antioxidant and oncogene rescue of metabolic defects caused by loss of matrix attachment. Nature.

[36] Irani K., Xia Y., Zweier J.L., Sollott S.J., Der C.J., Fearon E.R., Sundaresan M., Finkel T., Goldschmidt-Clermont P.J. (1997). Mitogenic signaling mediated by oxidants in Ras-transformed fibroblasts. Science.

[37] Chatterjee A., Mambo E., Sidransky D. (2006). Mitochondrial DNA mutations in human cancer. Oncogene.

[38] Park J.S., Sharma L.K., Li H., Xiang R., Holstein D., Wu J., Lechleiter J., Naylor S.L., Deng J.J., Lu J. (2009). A heteroplasmic, not homoplasmic, mitochondrial DNA mutation promotes tumorigenesis via alteration in reactive oxygen species generation and apoptosis. Hum. Mol. Genet..

[39] Sharma L.K., Fang H., Liu J., Vartak R., Deng J., Bai Y. (2011). Mitochondrial respiratory complex I dysfunction promotes tumorigenesis through ROS alteration and AKT activation. Hum. Mol. Genet..

[40] Woo D.K., Green P.D., Santos J.H., D’Souza A.D., Walther Z., Martin W.D., Christian B.E., Chandel N.S., Shadel G.S. (2012). Mitochondrial genome instability and ROS enhance intestinal tumorigenesis in APC(Min/+) mice. Am. J. Pathol..

[41] Orimo A., Gupta P.B., Sgroi D.C., Arenzana-Seisdedos F., Delaunay T., Naeem R., Carey V.J., Richardson A.L., Weinberg R.A. (2005). Stromal fibroblasts present in invasive human breast carcinomas promote tumor growth and angiogenesis through elevated SDF-1/CXCL12 secretion. Cell.

[42] Ostman A., Augsten M. (2009). Cancer-associated fibroblasts and tumor growth--bystanders turning into key players. Curr. Opin. Genet. Dev..

[43] Serini G., Gabbiani G. (1999). Mechanisms of myofibroblast activity and phenotypic modulation. Exp. Cell Res..

[44] Madar S., Goldstein I., Rotter V. (2013). ‘Cancer associated fibroblasts’—More than meets the eye. Trends Mol. Med..

[45] Madar S., Brosh R., Buganim Y., Ezra O., Goldstein I., Solomon H., Kogan I., Goldfinger N., Klocker H., Rotter V. (2009). Modulated expression of WFDC1 during carcinogenesis and cellular senescence. Carcinogenesis.

[46] Buganim Y., Madar S., Rais Y., Pomeraniec L., Harel E., Solomon H., Kalo E., Goldstein I., Brosh R., Haimov O. (2011). Transcriptional activity of ATF3 in the stromal compartment of tumors promotes cancer progression. Carcinogenesis.

[47] Rasmussen A.A., Cullen K.J. (1998). Paracrine/autocrine regulation of breast cancer by the insulin-like growth factors. Breast Cancer Res. Treat..

[48] Noel A., De Pauw-Gillet M.C., Purnell G., Nusgens B., Lapiere C.M., Foidart J.M. (1993). Enhancement of tumorigenicity of human breast adenocarcinoma cells in nude mice by matrigel and fibroblasts. Br. J. Cancer.

[49] Kuperwasser C., Chavarria T., Wu M., Magrane G., Gray J.W., Carey L., Richardson A., Weinberg R.A. (2004). Reconstruction of functionally normal and malignant human breast tissues in mice. Proc. Natl. Acad. Sci. USA.

[50] Gascard P., Tlsty T.D. (2016). Carcinoma-associated fibroblasts: Orchestrating the composition of malignancy. Genes Dev..

[51] Chauhan V.P., Boucher Y., Ferrone C.R., Roberge S., Martin J.D., Stylianopoulos T., Bardeesy N., DePinho R.A., Padera T.P., Munn L.L. (2014). Compression of pancreatic tumor blood vessels by hyaluronan is caused by solid stress and not interstitial fluid pressure. Cancer Cell.

[52] Jezierska-Drutel A., Rosenzweig S.A., Neumann C.A. (2013). Role of oxidative stress and the microenvironment in breast cancer development and progression. Adv. Cancer Res..

[53] Radisky E.S., Radisky D.C. (2007). Stromal induction of breast cancer: Inflammation and invasion. Rev. Endocr. Metab. Disord..

[54] Zavadil J., Haley J., Kalluri R., Muthuswamy S.K., Thompson E. (2008). Epithelial-mesenchymal transition. Cancer Res..

[55] Zeisberg E.M., Potenta S., Xie L., Zeisberg M., Kalluri R. (2007). Discovery of endothelial to mesenchymal transition as a source for carcinoma-associated fibroblasts. Cancer Res..

[56] McDonald L.T., Russell D.L., Kelly R.R., Xiong Y., Motamarry A., Patel R.K., Jones J.A., Watson P.M., Turner D.P., Watson D.K. (2015). Hematopoietic stem cell-derived cancer-associated fibroblasts are novel contributors to the pro-tumorigenic microenvironment. Neoplasia.

[57] Dirat B., Bochet L., Dabek M., Daviaud D., Dauvillier S., Majed B., Wang Y.Y., Meulle A., Salles B., Le Gonidec S. (2011). Cancer-associated adipocytes exhibit an activated phenotype and contribute to breast cancer invasion. Cancer Res..

[58] Kojima Y., Acar A., Eaton E.N., Mellody K.T., Scheel C., Ben-Porath I., Onder T.T., Wang Z.C., Richardson A.L., Weinberg R.A. (2010). Autocrine TGF-beta and stromal cell-derived factor-1 (SDF-1) signaling drives the evolution of tumor-promoting mammary stromal myofibroblasts. Proc. Natl. Acad. Sci. USA.

[59] Mueller L., Goumas F.A., Affeldt M., Sandtner S., Gehling U.M., Brilloff S., Walter J., Karnatz N., Lamszus K., Rogiers X. (2007). Stromal fibroblasts in colorectal liver metastases originate from resident fibroblasts and generate an inflammatory microenvironment. Am. J. Pathol..

[60] Toullec A., Gerald D., Despouy G., Bourachot B., Cardon M., Lefort S., Richardson M., Rigaill G., Parrini M.C., Lucchesi C. (2010). Oxidative stress promotes myofibroblast differentiation and tumour spreading. EMBO Mol. Med..

[61] Jain M., Rivera S., Monclus E.A., Synenki L., Zirk A., Eisenbart J., Feghali-Bostwick C., Mutlu G.M., Budinger G.R., Chandel N.S. (2013). Mitochondrial reactive oxygen species regulate transforming growth factor-beta signaling. J. Biol. Chem..

[62] Artaud-Macari E., Goven D., Brayer S., Hamimi A., Besnard V., Marchal-Somme J., Ali Z.E., Crestani B., Kerdine-Romer S., Boutten A. (2013). Nuclear factor erythroid 2-related factor 2 nuclear translocation induces myofibroblastic dedifferentiation in idiopathic pulmonary fibrosis. Antioxid. Redox Signal..

[63] Sampson N., Koziel R., Zenzmaier C., Bubendorf L., Plas E., Jansen-Durr P., Berger P. (2011). ROS signaling by NOX4 drives fibroblast-to-myofibroblast differentiation in the diseased prostatic stroma. Mol. Endocrinol..

[64] Frijhoff J., Dagnell M., Augsten M., Beltrami E., Giorgio M., Ostman A. (2014). The mitochondrial reactive oxygen species regulator p66Shc controls PDGF-induced signaling and migration through protein tyrosine phosphatase oxidation. Free Radic. Biol. Med..

[65] Dagnell M., Frijhoff J., Pader I., Augsten M., Boivin B., Xu J., Mandal P.K., Tonks N.K., Hellberg C., Conrad M. (2013). Selective activation of oxidized PTP1B by the thioredoxin system modulates PDGF-beta receptor tyrosine kinase signaling. Proc. Natl. Acad. Sci. USA.

[66] Meng T.C., Fukada T., Tonks N.K. (2002). Reversible oxidation and inactivation of protein tyrosine phosphatases in vivo. Mol. Cell..

[67] Sundaresan M., Yu Z.X., Ferrans V.J., Irani K., Finkel T. (1995). Requirement for generation of H_2_O_2_ for platelet-derived growth factor signal transduction. Science.

[68] Salmeen A., Park B.O., Meyer T. (2010). The NADPH oxidases NOX4 and DUOX2 regulate cell cycle entry via a p53-dependent pathway. Oncogene.

[69] Sotgia F., Martinez-Outschoorn U.E., Howell A., Pestell R.G., Pavlides S., Lisanti M.P. (2012). Caveolin-1 and cancer metabolism in the tumor microenvironment: Markers, models, and mechanisms. Annu. Rev. Pathol..

[70] Martinez-Outschoorn U.E., Pavlides S., Whitaker-Menezes D., Daumer K.M., Milliman J.N., Chiavarina B., Migneco G., Witkiewicz A.K., Martinez-Cantarin M.P., Flomenberg N. (2010). Tumor cells induce the cancer associated fibroblast phenotype via caveolin-1 degradation: Implications for breast cancer and DCIS therapy with autophagy inhibitors. Cell Cycle.

[71] Martinez-Outschoorn U.E., Whitaker-Menezes D., Lin Z., Flomenberg N., Howell A., Pestell R.G., Lisanti M.P., Sotgia F. (2011). Cytokine production and inflammation drive autophagy in the tumor microenvironment: Role of stromal caveolin-1 as a key regulator. Cell Cycle.

[72] Martinez-Outschoorn U.E., Lin Z., Trimmer C., Flomenberg N., Wang C., Pavlides S., Pestell R.G., Howell A., Sotgia F., Lisanti M.P. (2011). Cancer cells metabolically “fertilize” the tumor microenvironment with hydrogen peroxide, driving the Warburg effect: Implications for PET imaging of human tumors. Cell Cycle.

[73] Martinez-Outschoorn U.E., Sotgia F., Lisanti M.P. (2015). Caveolae and signalling in cancer. Nat. Rev. Cancer.

[74] Martinez-Outschoorn U.E., Trimmer C., Lin Z., Whitaker-Menezes D., Chiavarina B., Zhou J., Wang C., Pavlides S., Martinez-Cantarin M.P., Capozza F. (2010). Autophagy in cancer associated fibroblasts promotes tumor cell survival: Role of hypoxia, HIF1 induction and NFkappaB activation in the tumor stromal microenvironment. Cell Cycle.

[75] Asterholm I.W., Mundy D.I., Weng J., Anderson R.G., Scherer P.E. (2012). Altered mitochondrial function and metabolic inflexibility associated with loss of caveolin-1. Cell Metab..

[76] Svensson K.J., Christianson H.C., Wittrup A., Bourseau-Guilmain E., Lindqvist E., Svensson L.M., Morgelin M., Belting M. (2013). Exosome uptake depends on ERK1/2-heat shock protein 27 signaling and lipid Raft-mediated endocytosis negatively regulated by caveolin-1. J. Biol. Chem..

[77] Zhao H., Achreja A., Iessi E., Logozzi M., Mizzoni D., Di Raimo R., Nagrath D., Fais S. (2017). The key role of extracellular vesicles in the metastatic process. Biochim. Biophys. Acta.

[78] Cho J.A., Park H., Lim E.H., Lee K.W. (2012). Exosomes from breast cancer cells can convert adipose tissue-derived mesenchymal stem cells into myofibroblast-like cells. Int. J. Oncol..

[79] Paggetti J., Haderk F., Seiffert M., Janji B., Distler U., Ammerlaan W., Kim Y.J., Adam J., Lichter P., Solary E. (2015). Exosomes released by chronic lymphocytic leukemia cells induce the transition of stromal cells into cancer-associated fibroblasts. Blood.

[80] Gangoda L., Boukouris S., Liem M., Kalra H., Mathivanan S. (2015). Extracellular vesicles including exosomes are mediators of signal transduction: Are they protective or pathogenic?. Proteomics.

[81] Zhao H., Yang L., Baddour J., Achreja A., Bernard V., Moss T., Marini J.C., Tudawe T., Seviour E.G., San Lucas F.A. (2016). Tumor microenvironment derived exosomes pleiotropically modulate cancer cell metabolism. Elife.

[82] Kroemer G., Marino G., Levine B. (2010). Autophagy and the integrated stress response. Mol. Cell..

[83] Yang Z., Klionsky D.J. (2010). Eaten alive: A history of macroautophagy. Nat. Cell Biol..

[84] Scherz-Shouval R., Shvets E., Fass E., Shorer H., Gil L., Elazar Z. (2007). Reactive oxygen species are essential for autophagy and specifically regulate the activity of Atg4. EMBO J..

[85] Carroll B., Otten E.G., Manni D., Stefanatos R., Menzies F.M., Smith G.R., Jurk D., Kenneth N., Wilkinson S., Passos J.F. (2018). Oxidation of SQSTM1/p62 mediates the link between redox state and protein homeostasis. Nat. Commun..

[86] Li L., Chen Y., Gibson S.B. (2013). Starvation-induced autophagy is regulated by mitochondrial reactive oxygen species leading to AMPK activation. Cell. Signal..

[87] Filomeni G., De Zio D., Cecconi F. (2015). Oxidative stress and autophagy: The clash between damage and metabolic needs. Cell Death Differ..

[88] Guo J.Y., Karsli-Uzunbas G., Mathew R., Aisner S.C., Kamphorst J.J., Strohecker A.M., Chen G., Price S., Lu W., Teng X. (2013). Autophagy suppresses progression of K-ras-induced lung tumors to oncocytomas and maintains lipid homeostasis. Genes Dev..

[89] Lan S.H., Wu S.Y., Zuchini R., Lin X.Z., Su I.J., Tsai T.F., Lin Y.J., Wu C.T., Liu H.S. (2014). Autophagy suppresses tumorigenesis of hepatitis B virus-associated hepatocellular carcinoma through degradation of microRNA-224. Hepatology.

[90] Liu X.D., Yao J., Tripathi D.N., Ding Z., Xu Y., Sun M., Zhang J., Bai S., German P., Hoang A. (2015). Autophagy mediates HIF2alpha degradation and suppresses renal tumorigenesis. Oncogene.

[91] Cianfanelli V., Fuoco C., Lorente M., Salazar M., Quondamatteo F., Gherardini P.F., De Zio D., Nazio F., Antonioli M., D’Orazio M. (2015). AMBRA1 links autophagy to cell proliferation and tumorigenesis by promoting c-Myc dephosphorylation and degradation. Nat. Cell Biol..

[92] Degenhardt K., Mathew R., Beaudoin B., Bray K., Anderson D., Chen G., Mukherjee C., Shi Y., Gelinas C., Fan Y. (2006). Autophagy promotes tumor cell survival and restricts necrosis, inflammation, and tumorigenesis. Cancer Cell.

[93] Capparelli C., Whitaker-Menezes D., Guido C., Balliet R., Pestell T.G., Howell A., Sneddon S., Pestell R.G., Martinez-Outschoorn U., Lisanti M.P. (2012). CTGF drives autophagy, glycolysis and senescence in cancer-associated fibroblasts via HIF1 activation, metabolically promoting tumor growth. Cell Cycle.

[94] Wang Q., Xue L., Zhang X., Bu S., Zhu X., Lai D. (2016). Autophagy protects ovarian cancer-associated fibroblasts against oxidative stress. Cell Cycle.

[95] Pavlides S., Vera I., Gandara R., Sneddon S., Pestell R.G., Mercier I., Martinez-Outschoorn U.E., Whitaker-Menezes D., Howell A., Sotgia F. (2012). Warburg meets autophagy: Cancer-associated fibroblasts accelerate tumor growth and metastasis via oxidative stress, mitophagy, and aerobic glycolysis. Antioxid. Redox Signal..

[96] Martinez-Outschoorn U.E., Pavlides S., Howell A., Pestell R.G., Tanowitz H.B., Sotgia F., Lisanti M.P. (2011). Stromal-epithelial metabolic coupling in cancer: Integrating autophagy and metabolism in the tumor microenvironment. Int. J. Biochem. Cell Biol..

[97] Vasievich E.A., Huang L. (2011). The suppressive tumor microenvironment: A challenge in cancer immunotherapy. Mol. Pharm..

[98] Cemerski S., Cantagrel A., Van Meerwijk J.P., Romagnoli P. (2002). Reactive oxygen species differentially affect T cell receptor-signaling pathways. J. Biol. Chem..

[99] Devadas S., Zaritskaya L., Rhee S.G., Oberley L., Williams M.S. (2002). Discrete generation of superoxide and hydrogen peroxide by T cell receptor stimulation: Selective regulation of mitogen-activated protein kinase activation and fas ligand expression. J. Exp. Med..

[100] Jackson S.H., Devadas S., Kwon J., Pinto L.A., Williams M.S. (2004). T cells express a phagocyte-type NADPH oxidase that is activated after T cell receptor stimulation. Nat. Immunol..

[101] Kaminski M.M., Sauer S.W., Kaminski M., Opp S., Ruppert T., Grigaravicius P., Grudnik P., Grone H.J., Krammer P.H., Gulow K. (2012). T cell activation is driven by an ADP-dependent glucokinase linking enhanced glycolysis with mitochondrial reactive oxygen species generation. Cell Rep..

[102] Kaminski M., Kiessling M., Suss D., Krammer P.H., Gulow K. (2007). Novel role for mitochondria: Protein kinase Ctheta-dependent oxidative signaling organelles in activation-induced T-cell death. Mol. Cell. Biol..

[103] Scharping N.E., Menk A.V., Moreci R.S., Whetstone R.D., Dadey R.E., Watkins S.C., Ferris R.L., Delgoffe G.M. (2016). The Tumor Microenvironment Represses T Cell Mitochondrial Biogenesis to Drive Intratumoral T Cell Metabolic Insufficiency and Dysfunction. Immunity.

[104] Siska P.J., Beckermann K.E., Mason F.M., Andrejeva G., Greenplate A.R., Sendor A.B., Chiang Y.J., Corona A.L., Gemta L.F., Vincent B.G. (2017). Mitochondrial dysregulation and glycolytic insufficiency functionally impair CD8 T cells infiltrating human renal cell carcinoma. JCI Insight.

[105] Ligtenberg M.A., Mougiakakos D., Mukhopadhyay M., Witt K., Lladser A., Chmielewski M., Riet T., Abken H., Kiessling R. (2016). Coexpressed Catalase Protects Chimeric Antigen Receptor-Redirected T Cells as well as Bystander Cells from Oxidative Stress-Induced Loss of Antitumor Activity. J. Immunol..

[106] Okazaki T., Maeda A., Nishimura H., Kurosaki T., Honjo T. (2001). PD-1 immunoreceptor inhibits B cell receptor-mediated signaling by recruiting src homology 2-domain-containing tyrosine phosphatase 2 to phosphotyrosine. Proc. Natl. Acad. Sci. USA.

[107] Li J., Jie H.B., Lei Y., Gildener-Leapman N., Trivedi S., Green T., Kane L.P., Ferris R.L. (2015). PD-1/SHP-2 inhibits Tc1/Th1 phenotypic responses and the activation of T cells in the tumor microenvironment. Cancer Res..

[108] Chamoto K., Chowdhury P.S., Kumar A., Sonomura K., Matsuda F., Fagarasan S., Honjo T. (2017). Mitochondrial activation chemicals synergize with surface receptor PD-1 blockade for T cell-dependent antitumor activity. Proc. Natl. Acad. Sci. USA.

[109] Scharping N.E., Menk A.V., Whetstone R.D., Zeng X., Delgoffe G.M. (2017). Efficacy of PD-1 Blockade is Potentiated by Metformin-Induced Reduction of Tumor Hypoxia. Cancer Immunol. Res..

[110] Zou W., Wolchok J.D., Chen L. (2016). PD-L1 (B7-H1) and PD-1 pathway blockade for cancer therapy: Mechanisms, response biomarkers, and combinations. Sci. Transl. Med..

[111] Robert C., Long G.V., Brady B., Dutriaux C., Maio M., Mortier L., Hassel J.C., Rutkowski P., McNeil C., Kalinka-Warzocha E. (2015). Nivolumab in previously untreated melanoma without BRAF mutation. N. Engl. J. Med..

[112] Reck M., Rodriguez-Abreu D., Robinson A.G., Hui R., Csoszi T., Fulop A., Gottfried M., Peled N., Tafreshi A., Cuffe S. (2016). Pembrolizumab versus Chemotherapy for PD-L1-Positive Non-Small-Cell Lung Cancer. N. Engl. J. Med..

[113] Delgoffe G.M., Kole T.P., Zheng Y., Zarek P.E., Matthews K.L., Xiao B., Worley P.F., Kozma S.C., Powell J.D. (2009). The mTOR kinase differentially regulates effector and regulatory T cell lineage commitment. Immunity.

[114] Zeng H., Yang K., Cloer C., Neale G., Vogel P., Chi H. (2013). mTORC1 couples immune signals and metabolic programming to establish T(reg)-cell function. Nature.

[115] Shi L.Z., Wang R., Huang G., Vogel P., Neale G., Green D.R., Chi H. (2011). HIF1alpha-dependent glycolytic pathway orchestrates a metabolic checkpoint for the differentiation of TH17 and Treg cells. J. Exp. Med..

[116] Dang E.V., Barbi J., Yang H.Y., Jinasena D., Yu H., Zheng Y., Bordman Z., Fu J., Kim Y., Yen H.R. (2011). Control of T(H)17/T(reg) balance by hypoxia-inducible factor 1. Cell.

[117] Maj T., Wang W., Crespo J., Zhang H., Wang W., Wei S., Zhao L., Vatan L., Shao I., Szeliga W. (2017). Oxidative stress controls regulatory T cell apoptosis and suppressor activity and PD-L1-blockade resistance in tumor. Nat. Immunol..

[118] Kunisada Y., Eikawa S., Tomonobu N., Domae S., Uehara T., Hori S., Furusawa Y., Hase K., Sasaki A., Udono H. (2017). Attenuation of CD4(+) CD25(+) Regulatory T Cells in the Tumor Microenvironment by Metformin, a Type 2 Diabetes Drug. EBioMedicine.

[119] Weinberg S.E., Singer B.D., Steinert E.M., Martinez C.A., Mehta M.M., Martinez-Reyes I., Gao P., Helmin K.A., Abdala-Valencia H., Sena L.A. (2019). Mitochondrial complex III is essential for suppressive function of regulatory T cells. Nature.

[120] Schieber M., Chandel N.S. (2014). ROS function in redox signaling and oxidative stress. Curr. Biol..

[121] Zielonka J., Joseph J., Sikora A., Hardy M., Ouari O., Vasquez-Vivar J., Cheng G., Lopez M., Kalyanaraman B. (2017). Mitochondria-Targeted Triphenylphosphonium-Based Compounds: Syntheses, Mechanisms of Action, and Therapeutic and Diagnostic Applications. Chem. Rev..

[122] Qu P., Boelte K.C., Lin P.C. (2012). Negative regulation of myeloid-derived suppressor cells in cancer. Immunol. Investig..

[123] OuYang L.Y., Wu X.J., Ye S.B., Zhang R.X., Li Z.L., Liao W., Pan Z.Z., Zheng L.M., Zhang X.S., Wang Z. (2015). Tumor-induced myeloid-derived suppressor cells promote tumor progression through oxidative metabolism in human colorectal cancer. J. Transl. Med..

[124] Wei J., Zhang M., Zhou J. (2015). Myeloid-derived suppressor cells in major depression patients suppress T-cell responses through the production of reactive oxygen species. Psychiatry Res..

[125] Liu Y., Wei J., Guo G., Zhou J. (2015). Norepinephrine-induced myeloid-derived suppressor cells block T-cell responses via generation of reactive oxygen species. Immunopharmacol. Immunotoxicol..

[126] Weinberg S.E., Sena L.A., Chandel N.S. (2015). Mitochondria in the regulation of innate and adaptive immunity. Immunity.

[127] Kraaij M.D., Savage N.D., van der Kooij S.W., Koekkoek K., Wang J., van den Berg J.M., Ottenhoff T.H., Kuijpers T.W., Holmdahl R., van Kooten C. (2010). Induction of regulatory T cells by macrophages is dependent on production of reactive oxygen species. Proc. Natl. Acad. Sci. USA.

[128] Lin X., Zheng W., Liu J., Zhang Y., Qin H., Wu H., Xue B., Lu Y., Shen P. (2013). Oxidative stress in malignant melanoma enhances tumor necrosis factor-alpha secretion of tumor-associated macrophages that promote cancer cell invasion. Antioxid. Redox Signal..

[129] Purcell R.V., Pearson J., Aitchison A., Dixon L., Frizelle F.A., Keenan J.I. (2017). Colonization with enterotoxigenic Bacteroides fragilis is associated with early-stage colorectal neoplasia. PLoS ONE.

[130] Wu S., Rhee K.J., Albesiano E., Rabizadeh S., Wu X., Yen H.R., Huso D.L., Brancati F.L., Wick E., McAllister F. (2009). A human colonic commensal promotes colon tumorigenesis via activation of T helper type 17 T cell responses. Nat. Med..

[131] Kostic A.D., Chun E., Robertson L., Glickman J.N., Gallini C.A., Michaud M., Clancy T.E., Chung D.C., Lochhead P., Hold G.L. (2013). Fusobacterium nucleatum potentiates intestinal tumorigenesis and modulates the tumor-immune microenvironment. Cell Host Microbe.

[132] Gur C., Ibrahim Y., Isaacson B., Yamin R., Abed J., Gamliel M., Enk J., Bar-On Y., Stanietsky-Kaynan N., Coppenhagen-Glazer S. (2015). Binding of the Fap2 protein of Fusobacterium nucleatum to human inhibitory receptor TIGIT protects tumors from immune cell attack. Immunity.

[133] Mangerich A., Knutson C.G., Parry N.M., Muthupalani S., Ye W., Prestwich E., Cui L., McFaline J.L., Mobley M., Ge Z. (2012). Infection-induced colitis in mice causes dynamic and tissue-specific changes in stress response and DNA damage leading to colon cancer. Proc. Natl. Acad. Sci. USA.

[134] Huycke M.M., Abrams V., Moore D.R. (2002). Enterococcus faecalis produces extracellular superoxide and hydrogen peroxide that damages colonic epithelial cell DNA. Carcinogenesis.

[135] Goodwin A.C., Destefano Shields C.E., Wu S., Huso D.L., Wu X., Murray-Stewart T.R., Hacker-Prietz A., Rabizadeh S., Woster P.M., Sears C.L. (2011). Polyamine catabolism contributes to enterotoxigenic Bacteroides fragilis-induced colon tumorigenesis. Proc. Natl. Acad. Sci. USA.

[136] Bernstein H., Bernstein C., Payne C.M., Dvorak K. (2009). Bile acids as endogenous etiologic agents in gastrointestinal cancer. World J. Gastroenterol..

[137] Devkota S., Wang Y., Musch M.W., Leone V., Fehlner-Peach H., Nadimpalli A., Antonopoulos D.A., Jabri B., Chang E.B. (2012). Dietary-fat-induced taurocholic acid promotes pathobiont expansion and colitis in Il10-/- mice. Nature.

[138] Jones R.M., Mercante J.W., Neish A.S. (2012). Reactive oxygen production induced by the gut microbiota: Pharmacotherapeutic implications. Curr. Med. Chem..

[139] Clark A., Mach N. (2017). The Crosstalk between the Gut Microbiota and Mitochondria during Exercise. Front. Physiol..

[140] Yardeni T., Tanes C.E., Bittinger K., Mattei L.M., Schaefer P.M., Singh L.N., Wu G.D., Murdock D.G., Wallace D.C. (2019). Host mitochondria influence gut microbiome diversity: A role for ROS. Sci. Signal..

[141] Gopalakrishnan V., Spencer C.N., Nezi L., Reuben A., Andrews M.C., Karpinets T.V., Prieto P.A., Vicente D., Hoffman K., Wei S.C. (2018). Gut microbiome modulates response to anti-PD-1 immunotherapy in melanoma patients. Science.

[142] Evans J.M., Donnelly L.A., Emslie-Smith A.M., Alessi D.R., Morris A.D. (2005). Metformin and reduced risk of cancer in diabetic patients. BMJ.

[143] Ota S., Horigome K., Ishii T., Nakai M., Hayashi K., Kawamura T., Kishino A., Taiji M., Kimura T. (2009). Metformin suppresses glucose-6-phosphatase expression by a complex I inhibition and AMPK activation-independent mechanism. Biochem. Biophys. Res. Commun..

[144] El-Mir M.Y., Nogueira V., Fontaine E., Averet N., Rigoulet M., Leverve X. (2000). Dimethylbiguanide inhibits cell respiration via an indirect effect targeted on the respiratory chain complex I. J. Biol. Chem..

[145] Owen M.R., Doran E., Halestrap A.P. (2000). Evidence that metformin exerts its anti-diabetic effects through inhibition of complex 1 of the mitochondrial respiratory chain. Biochem. J..

[146] Wheaton W.W., Weinberg S.E., Hamanaka R.B., Soberanes S., Sullivan L.B., Anso E., Glasauer A., Dufour E., Mutlu G.M., Budigner G.S. (2014). Metformin inhibits mitochondrial complex I of cancer cells to reduce tumorigenesis. Elife.

[147] Kurelac I., Umesh Ganesh N., Iorio M., Porcelli A.M., Gasparre G. (2019). The multifaceted effects of metformin on tumor microenvironment. Semin. Cell Dev. Biol..

[148] Stuart S.D., Schauble A., Gupta S., Kennedy A.D., Keppler B.R., Bingham P.M., Zachar Z. (2014). A strategically designed small molecule attacks alpha-ketoglutarate dehydrogenase in tumor cells through a redox process. Cancer Metab..

[149] Alistar A., Morris B.B., Desnoyer R., Klepin H.D., Hosseinzadeh K., Clark C., Cameron A., Leyendecker J., D’Agostino R., Topaloglu U. (2017). Safety and tolerability of the first-in-class agent CPI-613 in combination with modified FOLFIRINOX in patients with metastatic pancreatic cancer: A single-centre, open-label, dose-escalation, phase 1 trial. Lancet Oncol..

[150] Reisman S.A., Lee C.Y., Meyer C.J., Proksch J.W., Sonis S.T., Ward K.W. (2014). Topical application of the synthetic triterpenoid RTA 408 protects mice from radiation-induced dermatitis. Radiat. Res..

[151] Reisman S.A., Lee C.Y., Meyer C.J., Proksch J.W., Ward K.W. (2014). Topical application of the synthetic triterpenoid RTA 408 activates Nrf2 and induces cytoprotective genes in rat skin. Arch. Derm. Res..

[152] Reisman S.A., Goldsberry A.R., Lee C.Y., O’Grady M.L., Proksch J.W., Ward K.W., Meyer C.J. (2015). Topical application of RTA 408 lotion activates Nrf2 in human skin and is well-tolerated by healthy human volunteers. BMC Dermatol..

[153] Probst B.L., Trevino I., McCauley L., Bumeister R., Dulubova I., Wigley W.C., Ferguson D.A. (2015). RTA 408, A Novel Synthetic Triterpenoid with Broad Anticancer and Anti-Inflammatory Activity. PLoS ONE.

